# Correction: Endogenous Rab29 does not impact basal or stimulated LRRK2 pathway activity

**DOI:** 10.1042/BCJ20200458_COR

**Published:** 2025-05-22

**Keywords:** leucine rich repeat kinase, parkinson’s disease, rab GTPase

It has come to the attention of the authors of the article “Endogenous Rab29 does not impact basal or stimulated LRRK2 pathway activity” (DOI: 10.1042/BCJ20200458) that there is a duplication within Figure 2 – namely that the same data has been used for αLRRK2 (N-term) for both Figure 2B (lungs) and Figure 2C (spleen).

**Figure 2:**
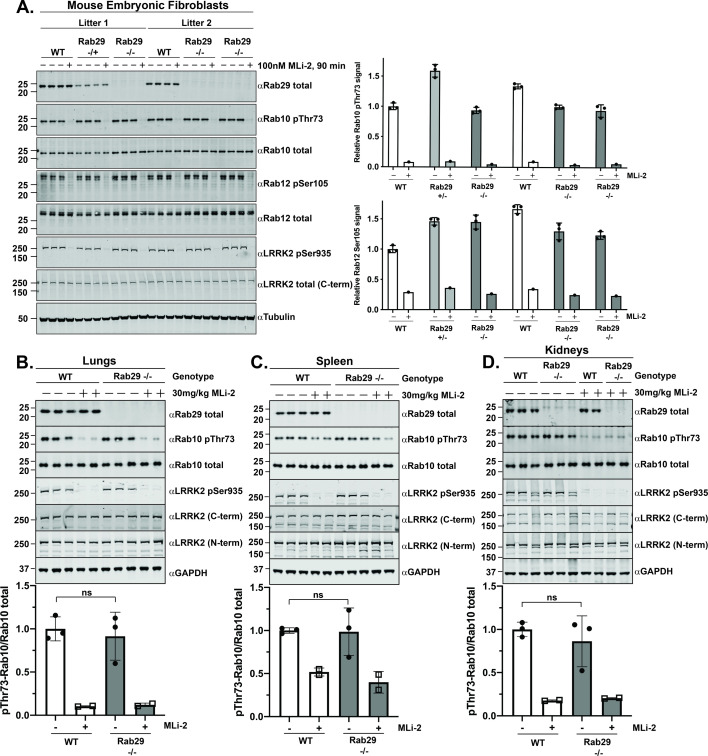
Knock-out of Rab29 does not affect basal LRRK2 activity (**A**) Two independent littermate-matched wildtype (WT), Rab29 knock-out heterozygous (+/-), or Rab29 knock-out homozygous (-/-) MEFs were treated with vehicle (DMSO) or 100 nM LRRK2 inhibitor MLi-2 for 90 min prior to harvest. Twenty micrograms of whole-cell extracts were subjected to quantitative immunoblot analysis with the indicated antibodies. Technical replicates represent cell extract obtained from a different dish of cells. The membranes were developed using the LI-COR Odyssey CLx western blot imaging system. Quantified data are presented as the mean ± SD of phospho-Rab10/total Rab10 and phospho-Rab12/total Rab12 ratios, which were quantified using the Image Studio software. Values were normalized to the average of Litter 1 wildtype MEFs treated with DMSO. Similar results were obtained in three independent experiments. (**B–D**) 6-month-old, littermate-matched wildtype (WT) and Rab29 knock-out (-/-) mice were administered with vehicle [40% (w/v) (2-hydroxypropyl)-β-cyclodextrin] or 30 mg/kg MLi-2 dissolved in vehicle by subcutaneous injection 2 h prior to tissue collection. Forty micrograms of tissue extracts were analyzed by quantitative immunoblot as described in (**A**). Each lane represents tissue extract derived from a different mouse. Quantified data are presented as mean ± SD and values were normalized to the average of the wildtype, vehicle-treated mice. Data were analyzed using two-tailed unpaired t-test and there was no statistical significance between WT and Rab29 knock-out mice. The resulting P-values from the unpaired t-tests are (**B**) lungs *P*=0.6585, (**C**) spleen *P*=0.9318, (**D**) kidneys *P*=0.4793.

Original Licor data for this experiment have been identified and assessed, and it has been confirmed that the correct image was used for Figure 2B (lungs) and an incorrect image was used for Figure 2C (spleen).

The raw data and requested correction have been assessed by and agreed with the Publisher. The authors apologise for the error and any inconvenience this may have caused. The data analysis and conclusions are not affected by the error.

A corrected Figure 2 is presented here.

