# Supplementary material for: Endogenous Rab29 does not impact basal or stimulated LRRK2 pathway activity
Source: Biochem J. 2020 Nov 27;477(22):4397–423. doi: 10.1042/BCJ20200458 (PMC7702304; doi:10.1042/BCJ20200458)

Supplementary Figures

Supplementary Figure 1. Knock-out of Rab29 does not affect LRRK2 Ser935 phosphorylation or LRRK2-mediated Rab10 phosphorylation in brain. (A) As in Figure 2 (B-D), 6-month-old, littermate-matched wildtype (WT) and Rab29 knock-out (-/-) mice were administered with vehicle (40% (w/v) (2-hydroxypropyl)-β-cyclodextrin) or 30 mg/kg MLi-2 dissolved in vehicle by subcutaneous injection 2 h prior to tissue collection. 40 μg of tissue extracts derived from 7 different brain sections were analyzed by quantitative immunoblot with the indicated antibodies. Each lane represents tissue extract derived from a different mouse. **(B)** Quantified data are presented as the mean ± SD of phospho-Rab10/Total Rab10 ratios and phospho-LRRK2/total LRRK2 ratios, and values were quantified using the Image Studio software.

Supplementary Figure 2. Overexpression of Rab29 in tissues from 3.5-month-old mice does not impact LRRK2-mediated Rab10 phosphorylation. **(A-E)** 3.5-month-old wildtype (WT), heterozygous (Tg Rab29 Het), and homozygous (Tg Rab29 Hom), transgenic Rab29 overexpressing mice were administered with vehicle (40% (w/v) (2-hydroxypropyl)-β-cyclodextrin) or 30 mg/kg MLi-2 dissolved in vehicle by subcutaneous injection 2 h prior to tissue collection. 40 μg of tissue extracts were subjected to quantitative immunoblot analysis with the indicated antibodies. The membranes were developed using the LI-COR Odyssey CLx Western Blot imaging system. Quantified data are presented as the phospho-Rab10/GAPDH ratios calculated using the Image Studio software. Values were normalized to the average of the wildtype, vehicle treated mice. Each lane represents a tissue sample derived from a different animal. Quantifications are presented as mean ± SD.

Supplementary Figure 3. Overexpression of Rab29 in MEFs or primary lung fibroblasts does not impact LRRK2-mediated Rab10 phosphorylation. **(A)** Littermate-matched wildtype (WT), heterozygous (Tg Rab29 Het), or homozygous (Tg Rab29 Hom), transgenic Rab29 overexpressing MEFs were treated with vehicle (DMSO) or 100 nM LRRK2 inhibitor MLi-2 for 90 min prior to harvest. 20 µg of whole cell extracts were subjected to quantitative immunoblot analysis with the indicated antibodies. Technical replicates represent cell extract obtained from a different dish of cells. The membranes were developed using the LI-COR Odyssey CLx Western Blot imaging system. Quantified data are presented as the mean ± SD of phospho-Rab10/total Rab10 and phospho-Rab12/total Rab12. Rab29 levels were quantified by calculating the ratio of total Rab29/GAPDH. Data quantifications were undertaken using the Image Studio software and values were normalized to the average of wildtype MEFs treated with DMSO. **(B)** Primary lung fibroblasts derived from 3 different wildtype (WT) mice and 2 different transgenic, homozygous Rab29 overexpressing (Tg Rab29 Hom) mice were treated with vehicle (DMSO) or 100 nM LRRK2 inhibitor MLi-2 for 90 min prior to harvest. 20 µg of whole cell extracts were subjected to quantitative immunoblot analysis with the indicated antibodies. Technical replicates represent cell extract obtained from a different dish of cells. Quantified data are presented as the mean ± SD of phospho-Rab10/total Rab10. Rab29 levels were quantified by the ratio of total Rab29/GAPDH. Data quantifications were undertaken using the Image Studio software and values were normalized to the average of wildtype primary lung fibroblasts treated with DMSO.

Supplementary Figure 4. Knock-out of Rab29 does not reduce elevated Rab10 phosphorylation in pathogenic LRRK2[R1441C] knock-in mice. (A-B) As in Figure 5 (B-D), the indicated 6-month-old matched mice were administered with vehicle (40% (w/v) (2-hydroxypropyl)-β-cyclodextrin) or 30 mg/kg MLi-2 dissolved in vehicle by subcutaneous injection 2 h prior to tissue collection. 40 μg of tissue extracts were subjected to quantitative immunoblot analysis with the indicated antibodies. The membranes were developed using the LI-COR Odyssey CLx Western Blot imaging system. Quantified data are presented as the ratios of phospho-Rab10/total Rab10, calculated with the Image Studio software. Quantifications are presented as mean ± SD, normalized to vehicle treated, wildtype animals. Each lane represents a tissue sample from a different animal. Data were analyzed by one-way ANOVA with Tukey's multiple comparisons test and no statistical significance was determined between the genotypes. Wildtype vs LRRK2[R1441C]: P = 0.1413 (A), P = 0.7459 (B). Wildtype vs Rab29 knock-out: P = 0.9455 (A), P = 0.7867 (B). LRRK2[R1441C] vs Rab29 knock-out LRRK2[R1441C]: P = 0.9973 (A), P = 0.9768 (B).

Supplementary Figure 5. Microsomal enrichment by fractionation of LRRK2[R1441C] knock-in and Rab29 knock-out LRRK2[R1441C] knock-in lungs. Lung tissues from mice of the indicated genotypes were subjected to enrichment by fractionation to produce total, cytosolic, crude mitochondrial and microsomal fractions. 40 μg of the total fractions and the equivalent volumes of the remaining fractions were subjected to quantitative immunoblot analysis with the indicated antibodies. Antibodies against PDI, VDAC1, and GAPDH were used as markers for microsomal, mitochondrial and cytosolic fractions, respectively. HEK293 cells expressing FLAG-LRRK2 R1441C treated with or without 100 nM MLi-2 for 90 minutes were run in parallel as a control to confirm that phosphorylation of Ser1292 is LRRK2-mediated. 40 μg of lung tissue lysates from LRRK2[R1441C] and Rab29 knock-out LRRK2[R1441C] mice that were processed using the standard lysis method outlined in this study, were also run in parallel. The membranes were developed using the LI-COR Odyssey CLx Western Blot imaging system.

Supplementary Figure 6. Rab29 knock-out does not reduce the enhanced LRRK2-mediated phosphorylation of Rab10 in VPS35[D620N] knock-in mice. (A-B) As in Figure 6 (B-D), the indicated 6-month-old matched mice were administered with vehicle (40% (w/v) (2-hydroxypropyl)-β-cyclodextrin) or 30 mg/kg MLi-2 dissolved in vehicle by subcutaneous injection 2 h prior to tissue collection. 40 μg of tissue extracts were subjected to quantitative immunoblot analysis with the indicated antibodies. The membranes were developed using the LI-COR Odyssey CLx Western Blot imaging system. Quantified data are presented as the ratios of phospho-Rab10/total Rab10, calculated using Image Studio software. Quantifications are presented as mean ± SD, normalized to vehicle treated, wildtype animals. Each lane represents a tissue sample from a different animal. Data were analyzed by one-way ANOVA with Tukey's multiple comparisons test and there was a statistically significant difference between wildtype and VPS35[D620N] large intestine samples (**P = 0.0072 (A)), but not between wildtype and VPS35[D620N] spinal cord (P = 0.8768 (B)). All other comparisons were not statistically significant. Wildtype vs Rab29 knock-out: P = 0.9092 (A), P = 0.8629 (B). VPS35[D620N] vs Rab29 knock-out VPS35[D620N]: P = 0.6516 (A), 0.3935 (B).

Supplementary Figure 7. Cation ionophores nigericin and monensin enhance Rab10 phosphorylation in A549 wildtype and Rab29 knock-out cells. (A-D) A549 wildtype or Rab29 knock-out cells were treated with the appropriate vehicle or **(A-B)** 2 μM nigericin and **(C-D)** 10 μM monensin for the indicated periods of time. Cells were treated with DMSO or 100 nM LRRK2 inhibitor MLi-2 for 90 minutes prior to harvest. 15-20 μg of cell extract was subjected to quantitative immunoblot analysis with the indicated antibodies. The membranes were developed using the LI-COR Odyssey CLx Western Blot imaging system. Quantified data are presented as the ratios of phospho-Rab10/total Rab10, calculated using Image Studio software. Quantifications are presented as mean ± SD, normalized to the average of vehicle treated cells. Data were analyzed by one-way ANOVA with Dunnett's multiple comparisons test and there was a statistically significant difference in LRRK2-mediated Rab10 phosphorylation in **(A)** between control and 2h treatment (****P < 0.0001), control and 4h treatment (****P < 0.0001), control and 8h treatment (****P < 0.0001), and control and 16h treatment (****P < 0.0001). There was a statistically significant difference in Rab10 phosphorylation in **(B)** between control and 2h treatment (***P = 0.0001), control and 4h treatment (****P < 0.0001), control and 8h treatment (****P < 0.0001), and control and 16h treatment (****P < 0.0001). There was a statistically significant difference in Rab10 phosphorylation in **(C)** between control and 2h treatment (****P < 0.0001), control and 4h treatment (**P = 0.0039), control and 8h treatment (****P < 0.0001), and control and 16h treatment (****P < 0.0001). There was a statistically significant difference in Rab10 phosphorylation in **(D)** between control and 2h treatment (*P = 0.0165), control and 4h treatment (****P < 0.0001), control and 8h treatment (****P < 0.0001), and control and 16h treatment (***P = 0.0001). Similar results were obtained in three independent experiments for each agonist.

Supplementary Figure 8. Cation ionophores nigericin and monensin, and lysosomal stressors chloroquine and LLOMe enhance LRRK2-mediated Rab10 and Rab12 phosphorylation in wildtype and Rab29 knock-out MEFs. (A-E) As in Fig 8, Littermate matched wildtype and Rab29 knock-out MEFs were treated with vehicle or **(A)** 2 μM nigericin, **(B)** 10 μM monensin, **(C)** 50 μM chloroquine, **(D)** 100 μM chloroquine or **(E)** 1 mM LLOMe for the indicated periods of time. Cells were treated with DMSO or 100 nM LRRK2 inhibitor MLi-2 for 2 hours prior to harvest. 15-20 μg of cell extract was subjected to quantitative immunoblot analysis with the indicated antibodies. The membranes were developed using the LI-COR Odyssey CLx Western Blot imaging system. Quantified data are presented as the ratios of phospho-Rab10/total Rab10 and phospho-Rab12/total Rab12, which were calculated using the Image Studio software. Quantifications are presented as mean ± SD and were normalized to the average of the vehicle-treated cells for each respective genotype. Graphs are representative of two independent experiments, including the data presented in Fig 8. Data were analyzed by one-way ANOVA with Dunnett's multiple comparisons test to determine statistical significance between control and treated samples of each genotype. The treatment time points between wildtype and Rab29 knock-out MEFs were compared using two-tailed unpaired t-tests There was a statistically significant difference in LRRK2-mediated Rab10 phosphorylation between **(A)** wildtype control and 2h treatment (**P = 0.0086), wildtype control and 4h treatment (**P = 0.0017), wildtype control and 8h treatment (****P = 0.0001), and between Rab29 knock-out control and 2h, 4h, and 8h treatments (****P = 0.0001). There was a statistically significant difference in LRRK2-mediated Rab12 phosphorylation between **(A)** wildtype control and 2h treatment

(**P = 0.0048), wildtype control and 4h treatment (**P = 0.0014), wildtype control and 8h treatment (**P = 0.0018), and between Rab29 knock-out control and 2h, 4h, and 8h treatments (****P = 0.0001). There was no statistical significance between genotypes at each time point in **(A)** pRab10 Thr73/Rab10 total 2h P = 0.4036, 4h P = 0.1148, 8h P = 0.4901 or **(A)** pRab12 Ser105/Rab12 total 2h P = 0.8130, 4h = 0.3851, 8h = 0.4486. There was a statistically significant difference in LRRK2-mediated Rab10 phosphorylation between **(B)** wildtype control and 2h, 4h, and 8h treatments (****P = 0.0001), and between Rab29 knock-out control and 2h treatment (**P = 0.0043), Rab29 knock-out control and 4h treatment (***P = 0.0007), and Rab29 knock-out control and 8h treatment (***P = 0.0007). There was a statistically significant difference in LRRK2-mediated Rab12 phosphorylation between **(B)** wildtype control and 2h treatment (**P = 0.0059), wildtype control and 4h treatment (*P = 0.0423), wildtype control and 8h treatment (**P = 0.0023), and between Rab29 knock-out control and 2h, 4h, and 8h treatments (****P = 0.0001). There was no statistical significance between genotypes at each time point in **(B)** pRab10 Thr73/Rab10 total 2h P = 0.0618, 4h P = 0.4045, 8h P = 0.8062 or **(B)** pRab12 Ser105/Rab12 total 2h P = 0.7816, 4h = 0.2533, 8h = 0.0544. There was a statistically significant difference in LRRK2-mediated Rab10 phosphorylation between **(C)** wildtype control and 4h treatment (*P = 0.0196), wildtype control and 8h treatment (*P = 0.0160), and between Rab29 knock-out control and 4h treatment (**P = 0.0061), and Rab29 knock-out control and 8h treatment (***P = 0.0002). There was a statistically significant difference in LRRK2-mediated Rab12 phosphorylation between **(C)** wildtype control and 2h treatment (**P = 0.0025), wildtype control and 4h treatment (***P = 0.0006), wildtype control and 8h treatment (***P = 0.0003), and between Rab29 knock-out control and 2h treatment (**P = 0.0026) and Rab29 knock-out control and 4h and 8h treatments (****P = 0.0001). There was statistical significance between genotypes at **(C)** 4h time point pRab12 Ser105/Rab12 total *P = 0.0335, and 8h time point pRab10 Thr73/Rab10 total *P = 0.0394 and pRab12 Ser105/Rab12 total *P = 0.0117. All other comparisons in **(C)** were not significant (wildtype control and 2h treatment pRab10 Thr73/Rab10 total P = 0.0695, Rab29 knock-out control and 2h treatment pRab10 Thr73/Rab10 total P = 0.1668, pRab10 Thr73/Rab10 total 2h time point comparison P = 0.6570, pRab10 Thr73/Rab10 total 4h time point comparison P = 01023, pRab12 Ser105/Rab12 total 2h hour time point comparison P = 0.7947). There was a statistically significant difference in LRRK2-mediated Rab10 phosphorylation between **(E)** Rab29 knock-out control and 1h and 2h treatments (****P = 0.0001). There was a statistically significant difference in LRRK2-mediated Rab12 phosphorylation between **(E)** wildtype control and 1h treatment (**P = 0.0020) and wildtype control and 2h treatment (****P = 0.0001), and between Rab29 knock-out control and 2h treatment (**P = 0.0069). All other comparisons were not statistically significant (wildtype control and 1h treatment pRab10 Thr73/Rab10 total P = 0.4558, wildtype control and 2h treatment pRab10 Thr73/Rab10 total P = 0.1747, and Rab29 knock-out control and 1h treatment pRab12 Ser105/Rab12 total P = 0.1730). There was no statistical significance between genotypes at each time point in **(E)** pRab10 Thr73/Rab10 total 1h P = 0.5209, 2h P = 0.6733 or **(E)** pRab12 Ser105/Rab12 total 1h P = 0.2421, 2h = 0.3869.

Supplementary Figure 1

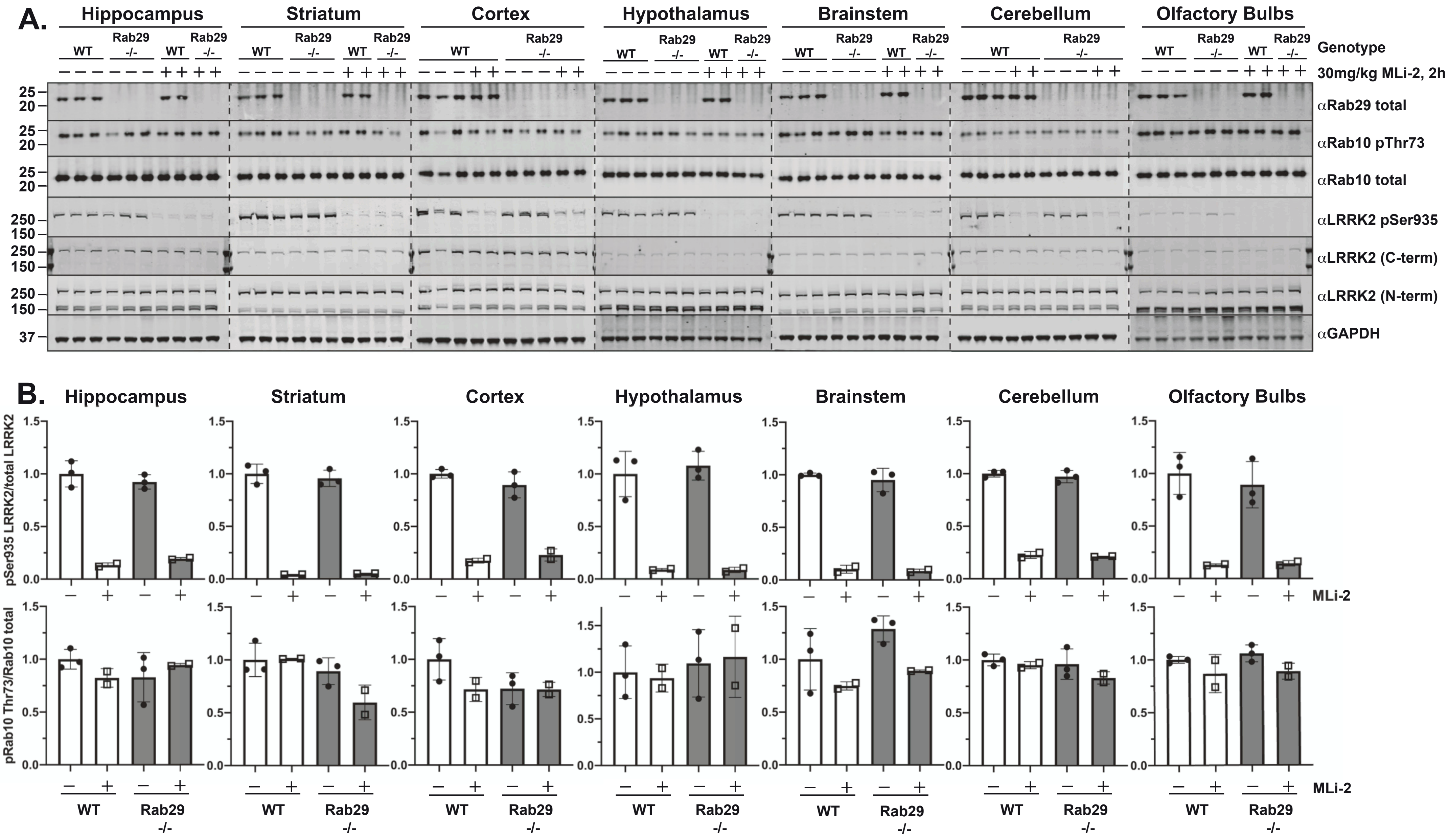

Supplementary Figure 2

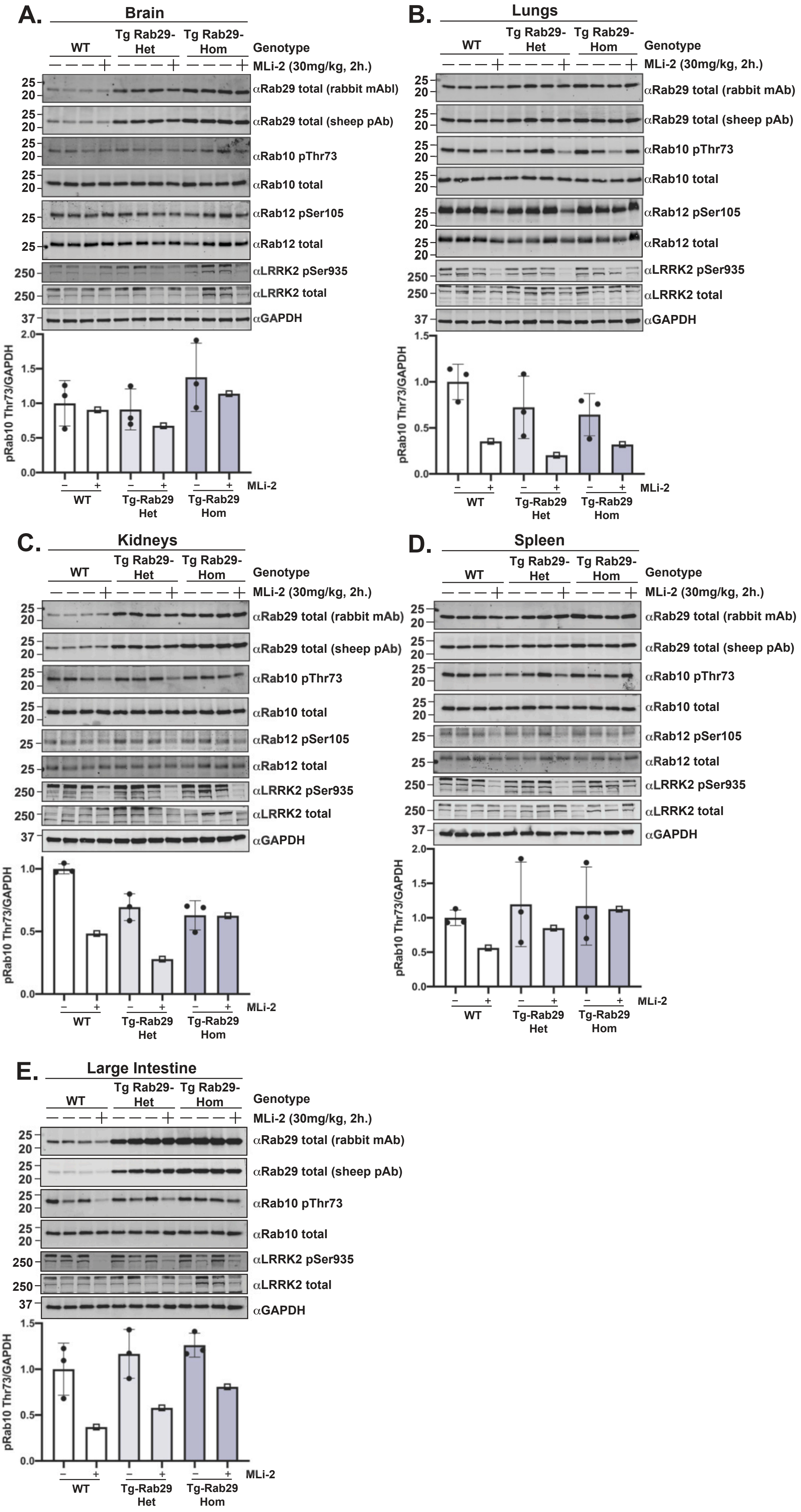

Supplementary Figure 3

A.

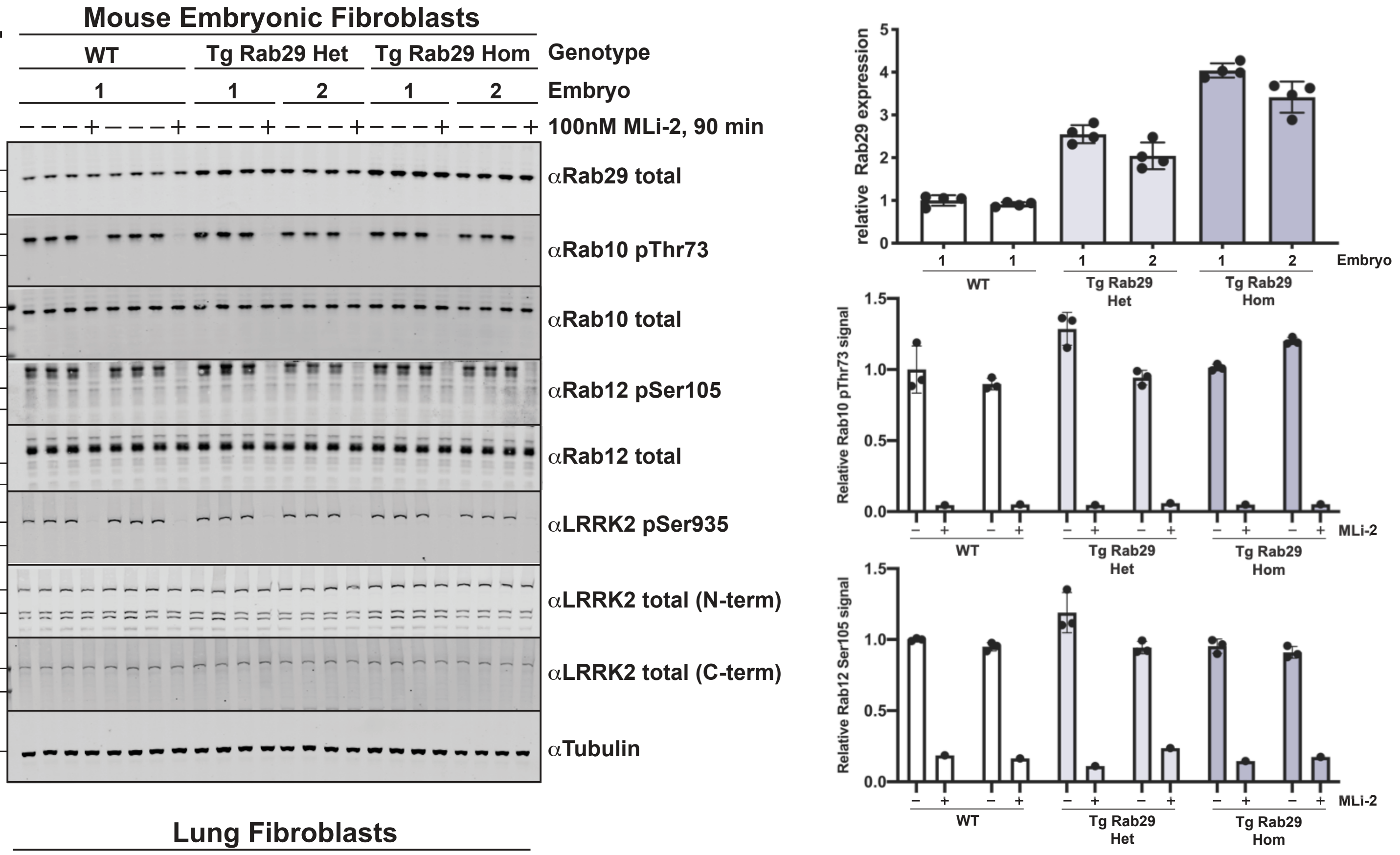

B.

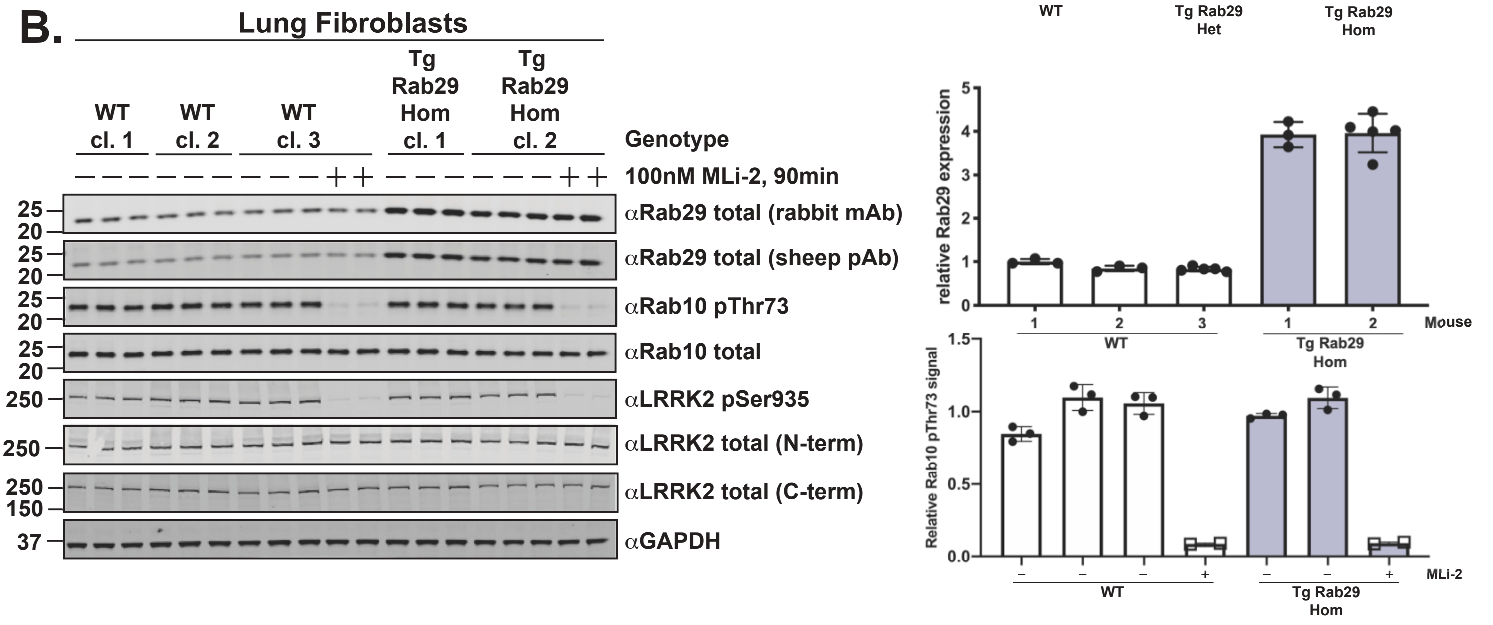

Supplementary Figure 4

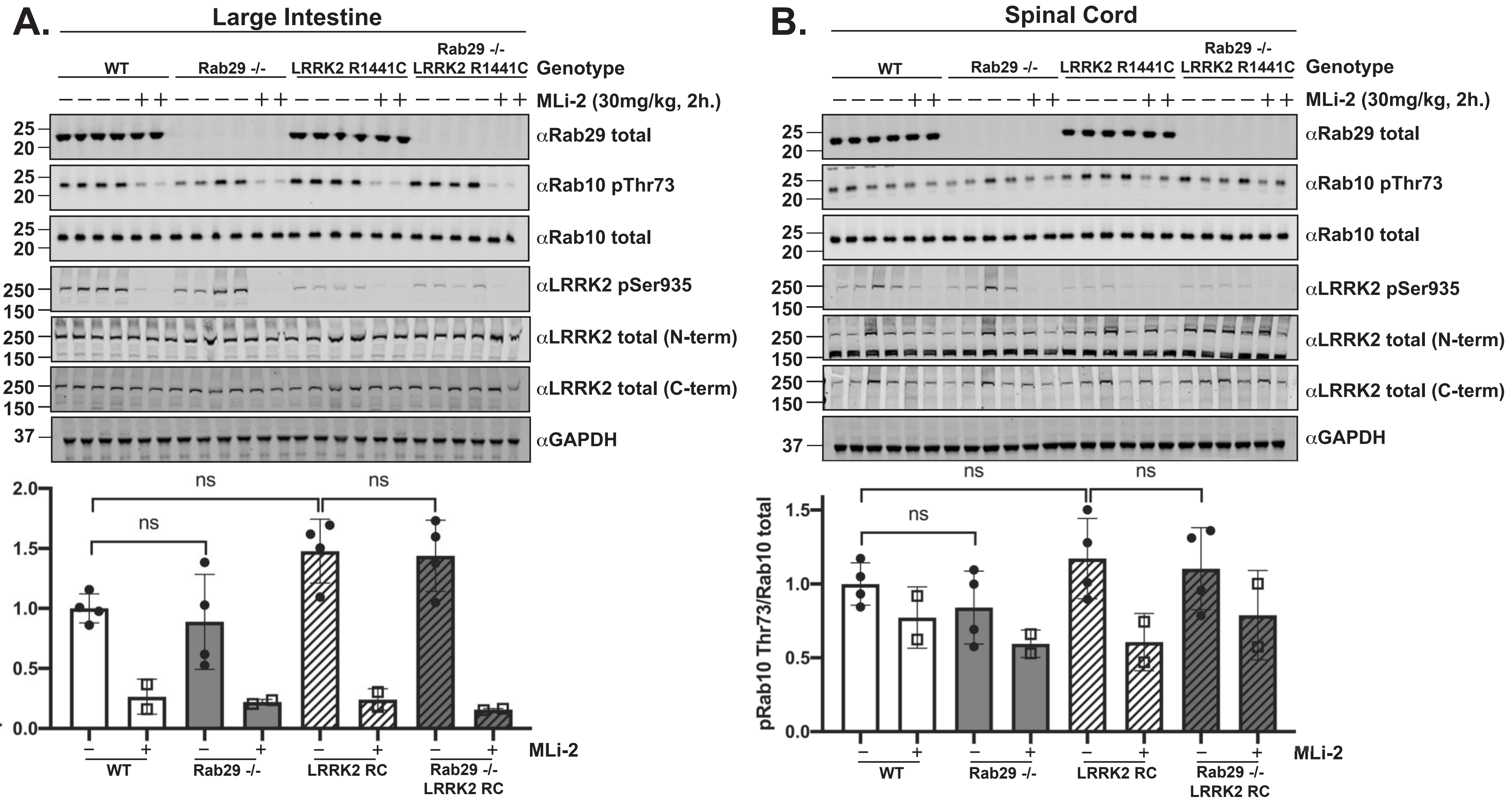

Supplementary Figure 5

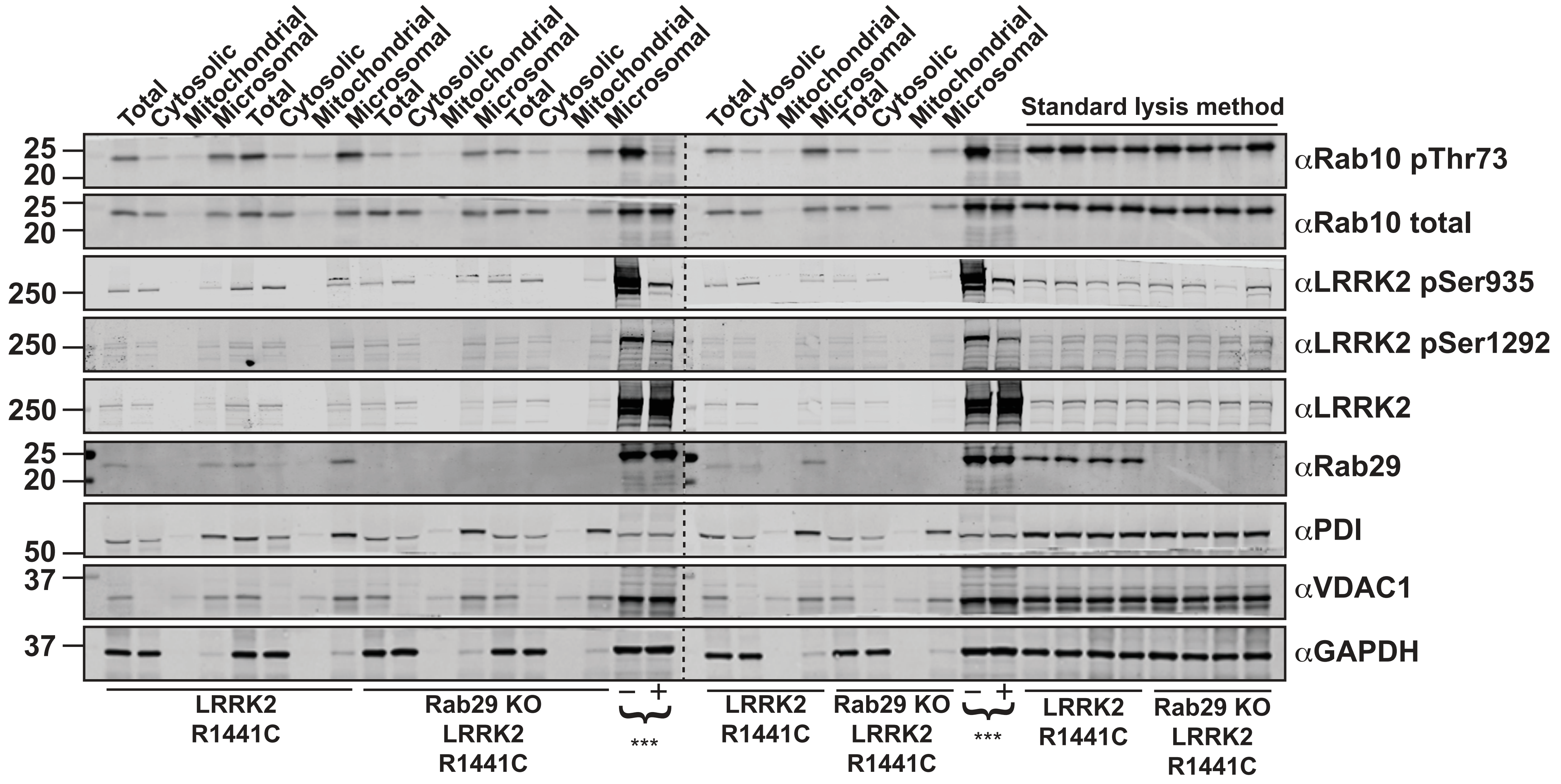

***HEK293 cells expressing LRRK2 R1441C treated with DMSO (-) or 100 nM MLi-2 (+) for 90 minutes

Supplementary Figure 6

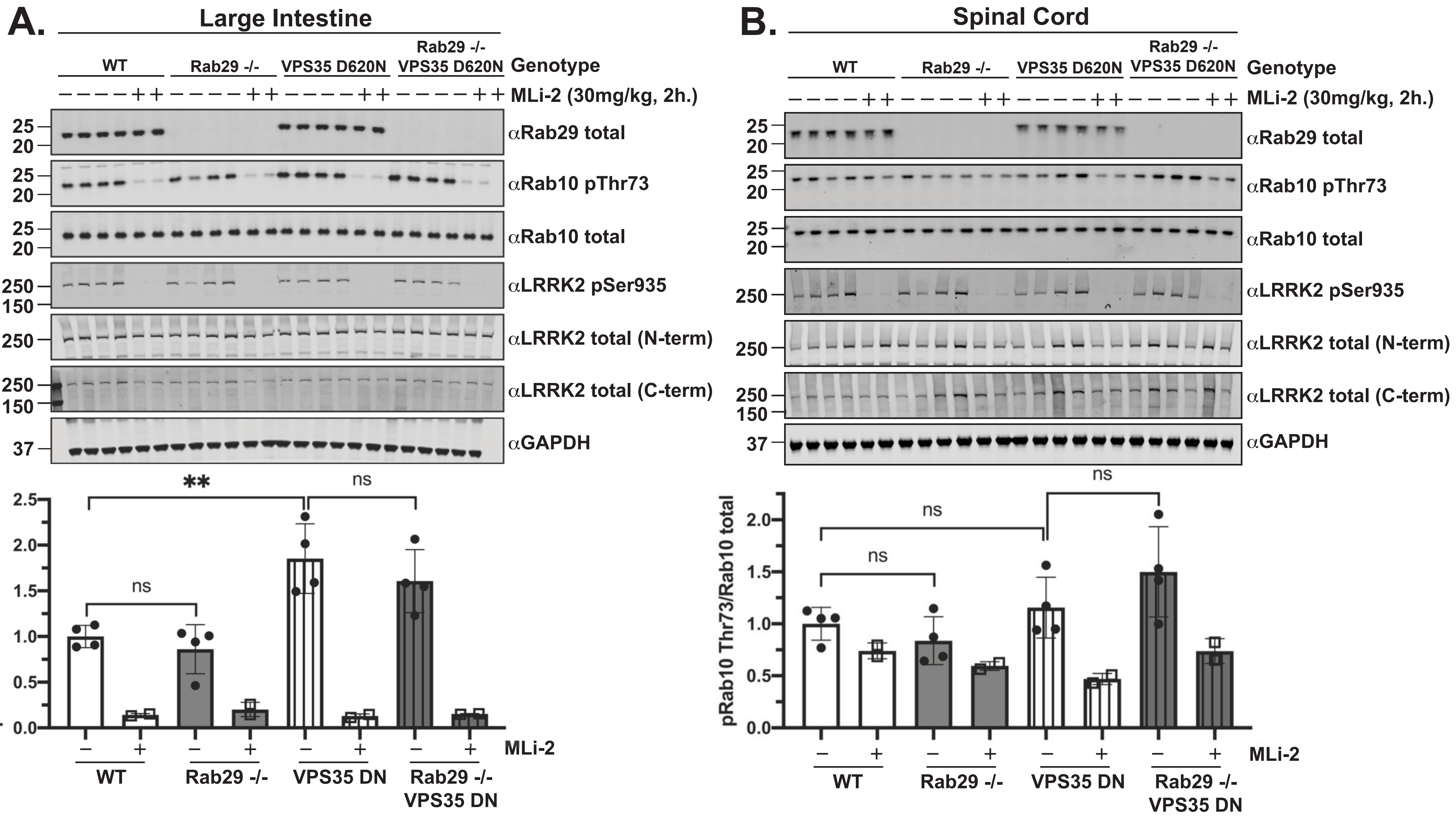

Supplementary Figure 7

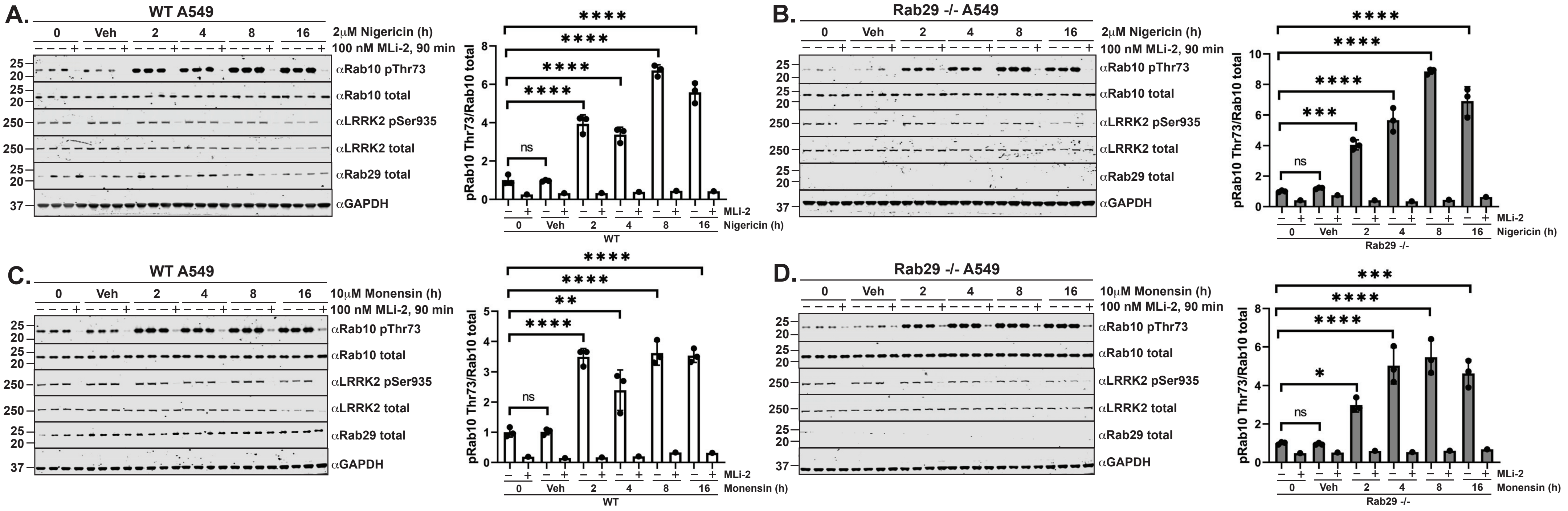

Supplementary Figure 8

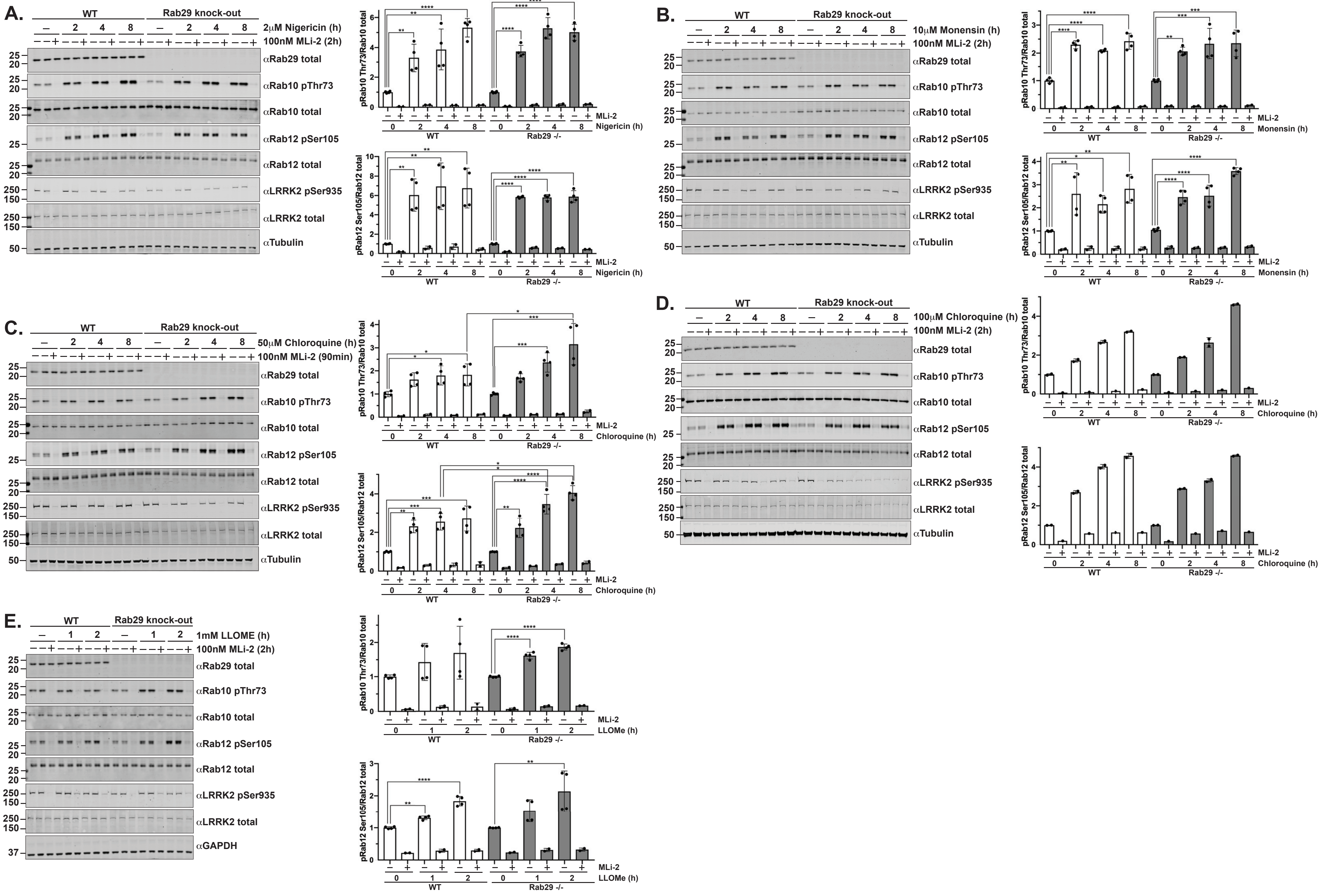

Supplement: Supplementary Figures S1-S8 [file BCJ-477-4397-s1.pdf]
